# The Potential Therapeutic Role of miR-223 in Bovine Endometritis by Targeting the NLRP3 Inflammasome

**DOI:** 10.3389/fimmu.2018.01916

**Published:** 2018-08-22

**Authors:** Gan Zhao, Kangfeng Jiang, Yaping Yang, Tao Zhang, Haichong Wu, Aftab Shaukat, Changwei Qiu, Ganzhen Deng

**Affiliations:** Department of Clinical Veterinary Medicine, College of Veterinary Medicine, Huazhong Agricultural University, Wuhan, China

**Keywords:** endometritis, NF-κB, MiR-223, NLRP3, inflammasome

## Abstract

Bovine endometritis affects milk production and reproductive performance in dairy cows and causes serious economic loss. The underlying molecular mechanisms or signaling pathways of bovine endometritis remain unclear. In this study, we attempted to determine the expression mechanism of mir-223 in endometritis of dairy cows and evaluate its potential therapeutic value. We first confirmed that there was an increased level of miR-223 in endometritis, and then, an LPS-induced bovine endometrial epithelial cell (BEND) line was used to mimic the inflammatory model *in vitro*. Our data showed that activation of NF-κB promoted the transcription of miR-223, thus inhibiting activation of the inflammatory mediator NLRP3 and its mediation of IL-1β production to protect against inflammatory damage. Meanwhile, *in vivo* studies showed that inhibition of mir-223 resulted in an enhanced pathology of mice during LPS-induced endometritis, while overexpression of mir-223 attenuated the inflammatory conditions in the uterus. In summary, our study highlights that miR-223 serves both to constrain the level of NLRP3 activation and to act as a protective factor in the inflammatory response and thus provides a future novel therapeutic modality for active flares in cow endometritis and other inflammatory diseases.

## Introduction

Uterine disorders, such as endometritis, are commonly observed in high-producing dairy cows and often cause poor reproductive performance, reduced milk yield, and consequently, result in economic loss (1–3). The endometrium is a natural barrier, and endometrial epithelial cells can resist the invasion of foreign pathogens by regulating the immune inflammatory response (4). Escherichia coli (*E. coli*), a Gram-negative bacterium, is one of the major pathogens that cause bovine endometritis via the production of the endotoxin lipopolysaccharide (LPS) (5, 6). As the main component of Gram-negative bacteria cell walls, LPS has strong biological activity that causes a strong systemic inflammatory response through the blood circulation (7, 8).

NOD-like receptor NLRP3 inflammasome is a cytoplasmic protein complex that consists of NLRP3, ASC (apoptosis-associated speck-like protein, which contains a caspase recruitment domain) and caspase-1, which has been shown to be a crucial regulator of diverse inflammatory diseases, including type 2 diabetes, atherosclerosis, and inflammatory bowel diseases (9, 10). NLRP3 plays critical roles in the initiation of inflammation and the development of immune responses and can be activated by a diverse series of endogenous and exogenous agonists; among them, LPS acts as the “priming” signal to induce subsequent activation processes (10, 11). The activation of the NLRP3 inflammasome triggers caspase-1 activation and, subsequently, leads to the processing of interleukin-1β (IL-1β), which contributes to an exacerbated inflammatory response (12, 13). Thus, to ease the inflammatory process and reduce tissue damage, the activation of the NLRP3 inflammasome should be tightly controlled (12).

MicroRNAs (miRNAs) are a family of short (20–24 nt) non-coding RNAs that are involved in post-transcriptional regulation of gene expression in multicellular organisms by affecting both the stability and translation of mRNAs. Accumulated evidence shows that miRNAs are critical gene regulators in many cellular processes, including inflammation (14, 15). Recently, RNA sequencing data revealed a large number of miRNAs associated with endometritis in dairy cows (16, 17); among them, miR-223, which is a highly conserved miRNA among species, showed a high expression level in dairy cows with endometritis and was also found to be as a crucial regulator in many inflammatory diseases, such as inflammatory bowel diseases (9) and rheumatoid arthritis (18). These results suggest that upregulation of miR-223 may be a novel biomarker in subsets of dairy cows with endometritis. Nuclear transcription factor (NF)-κB is activated in inflammation processes by many factors, including LPS and, subsequently, induces the transcription of downstream pro-inflammatory molecules, such as IL-1β, tumor necrosis factor-α (TNF-α) and IL-6(8). Recent reports have demonstrated that some miRNAs, such as miR-155, severe as targets of NF-κB, which in turn, diminish the production of inflammatory cytokines (19). Yang et al. and Kumar et al. showed that miR-223 contains a putative NF-κB binding site (20). Therefore, we sought to determine whether the activation of NF-upregulated miR-223 in dairy cows with endometritis. Moreover, nanoparticle delivery of a miR-223 mimetic *in vivo* significantly attenuated inflammation by targeting NLRP3 (9). These results indicate that miR-223 may act as an “anti-inflammatory signal” that protects the body against inflammatory damage. Nevertheless, there are limited mechanistic studies on how miR-223-regulated gene circuits shape endometritis. Thus, in the present study, we assume that NF-κB-induced upregulated miR-223 during endometritis may serve as a “protective factor” that inhibits the activation of the NLRP3 inflammasome, which may also a potential therapeutic target for the treatment of endometritis in dairy cows and other mammals.

## Materials and methods

### Bovine uteri samples collection

Uteri from Holstein cows were obtained from a local slaughter house. Healthy (*n* = 5) or inflamed uteri (*n* = 5, with visible lesions) were collected within 30 min after exsanguination in accordance with protocols approved by the Huazhong Agricultural University Animal Care and Use Committee (Wuhan, China) and then immediately transported to the laboratory on ice.

### Cell culture

BEND cells were purchased from the American Type Culture Collection (ATCC® CRL-2398™) and cultured in Dulbecco's modified Eagle's medium (DMEM)/F-12 (HyClone, USA) with 10% FBS (fetal bovine serum, PAN, Germany). 293T cells were kindly provided by Dr. Chenyang Yi (State Key Laboratory of Agricultural Microbiology, Huazhong Agricultural University) and maintained in RPMI 1640 (Invitrogen, USA) with 10% FBS (PAN, Germany). BEND cells were treated with LPS (from *E. coli* O55:B5, Merck, Germany) alone or with other corresponding treatments. After the treatments, the cells were prepared for further studies.

### Quantitative real-time PCR

Total RNA was extracted by TRIzol (Invitrogen, USA) according to the manufacturer's recommendation and then reverse-transcribed into cDNA using the Reverse Transcriptase M-MLV (TaKaRa) and Hairpin-it™ microRNA qPCR Quantitation Kit (GenePharma, Shanghai, China). qPCR was performed using the SYBR® Select Master Mix kit and standard protocols of the Step One Plus Real-Time PCR System (Applied Biosystems, USA). U6 small nuclear RNA was used as an internal control for miRNAs, and the mRNA levels were normalized to glyceraldehyde 3-phosphate dehydrogenase (GAPDH). The 2^−ΔΔCt^ comparative method was used to analyze expression levels. The mRNA, miRNA and U6 primers are listed in Supplementary Tables 1, 2.

### Plasmid and luciferase assay

The reporter plasmids pmirGLO-NLRP3-3UTR, pmirGLO-NLRP3-3UTR-MUT, and pmirGLO-NC (Promega) were designed and constructed using GeneCreate (Wuhan, China). Luciferase assays were performed with the Dual-Luciferase Reporter Assay System (Promega) according to the manufacturer's instructions. 293T cells were co-transfected with agomiR-223, antagomiR-223, pmirGLO-NLRP3-3UTR, pmirGLO-NLRP3-3UTR-MUT, or pmirGLO-NC (Promega) 36 h before the assay. The cells were then lysed to measure luciferase activity according to the manufacturer's instructions.

### Cell transfection

The miRNAs agomiRs and antagomirs were purchased from GenePharma Company (Shanghai, China). BEND or 293T cells were transfected with 50 nM miRNA agomiRs or 100 nM antagomirs in 6-well plates with Lipofectamine 2000 (Invitrogen) according to the manufacturer's instructions. siRNAs (the sequences are shown in Supplementary Table 3) for NLRP3 (si-NLRP3) and the negative control (si-NC) were designed and synthesized by GenePharma Co. (Shanghai, China). The siRNAs were transfected into BEND cells at a final concentration of 100 nM using Lipofectamine 2000 (Invitrogen) according to the manufacturer's instructions.

### NF-κB p65 immunofluorescence

Cells were cultured on six-well chamber slides. At the time of harvest, the cells were fixed with 4% paraformaldehyde and then permeabilized with 0.01% Triton X-100 for 10 min. Then, the cells were treated with anti-p-NF-κB p65 (#3033, Cell Signaling Technology, USA). Nuclei were stained using DAPI. Fluorescent images were taken using an AX70 widefield microscope (Olympus, Japan).

### Mouse model and sampling

Six- to eight-week-old BALB/c mice were obtained from the Animal Experiment Center of Huazhong Agricultural University (Wuhan, China). Mice were housed at a constant temperature (23°C) and relative humidity (60%) with a fixed 12 h light:12 h dark cycle with free access to food and water. All experimental procedures involving animals and their care were approved by the Animal Welfare and Research Ethics Committee of Huazhong Agricultural University. The LPS-induced endometritis mouse model was developed out as described previously (21). Briefly, six- to eight-week-old BALB/c mice were administered equal amounts of LPS (1 mg/kg) on each side of the uterus under anesthesia, and the control group received equal volumes of a saline solution. Mice of each group were treated as indicated above and then killed *via* CO_2_ inhalation. Uterine tissues from each group were harvested and immersed in 4% paraformaldehyde. The remaining tissues were stored at −80°C for subsequent experiments.

### Histological analysis

Uterine tissues were harvested, immersed in 4% paraformaldehyde, embedded in paraffin, cut into 4 μm sections, stained with hematoxylin/eosin (H&E) and examined under a microscope (Olympus, Japan). Histology scoring was performed in a blinded fashion. A combined score of inflammatory cell infiltration and tissue damage was determined.

### Immunohistochemistry

Uterine tissues were fixed in 4% paraformaldehyde, embedded in paraffin, sectioned and stained with anti-TNF-α (#ab6671, Abcam, Shanghai, China). Quantitative analysis was conducted by quantifying the fluorescence intensity from at least five sections.

### Cytokine assays

Tissues were harvested and homogenized with phosphate buffered saline (PBS), and the supernatants were collected after centrifugation. The concentrations of cytokines in tissues were measured using ELISA kits for TNF-α, IL-1β, and IL-6 (Biolegend, Thermo, USA) according to the manufacturer's instructions.

### Western blot

Total proteins from tissues and cells were extracted according to the manufacturer's recommended protocol (Vazyme, Nanjing, China). The protein concentrations were determined using the BCA Protein Assay Kit (Vazyme, Nanjing, China). Samples with equal amounts of protein (50 μg) were fractionated on 10% SDS–polyacrylamide gels, transferred to polyvinylidene difluoride membranes and blocked in 5% skim milk in TBST for 1.5 h at 25 ± 1°C. The membranes were then incubated at 4°C overnight with 1:1,000 dilutions (v/v) of the primary antibodies [anti-NLRP3 (#ab214185, Abcam, Shanghai, China) anti-p-NF-κB p65, (#3033, Cell Signaling Technology, USA), anti-NF-κB p65 (#8242, Cell Signaling Technology, USA), and anti-IκBα (#9242, Cell Signaling Technology, USA)]. After washing the membranes with TBST, incubations with 1:4,000 dilutions (v/v) of secondary antibodies were conducted for 2 h at 25 ± 1°C. Protein expression was detected using an Enhanced Chemiluminescence Detection System (ImageQuant LAS 4000 mini, USA). β-Actin was used as the loading control.

### Statistical analysis

All statistical analyses in this study were performed with GraphPad Prism 5 (GraphPad InStat Software, CA, USA). The data are expressed as the means ± S.E.M. Student's *t*-test was used to assess statistical significance (^*^P < 0.05, ^**^P < 0.01).

## Results

### Increased miR-223 expression is correlated with endometritis in dairy cows

**Figure 1:**
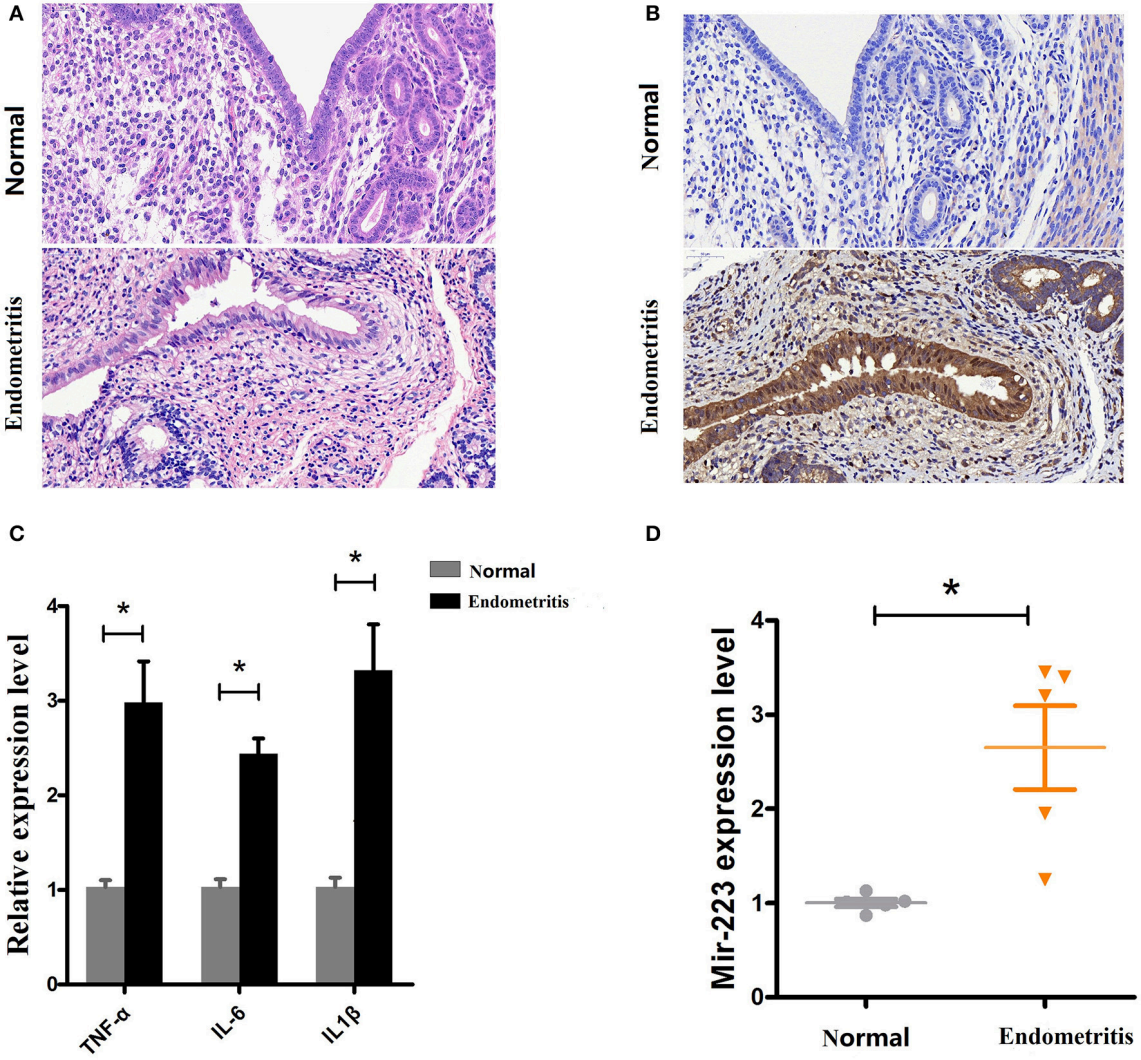
Expression of miR-223 in endometritis. **(A)** H&E staining of uterine tissue, scale bar, 50 μm. **(B)** Immunohistochemical staining of TNF-α, scale bar, 50 μm. **(C)** The mRNA levels of TNF-α, IL-1β, and IL-6 were measured by qPCR. **(D)** The miR-223 level in samples was detected by qPCR. Normal and endometritis stand for healthy uterus and uterus with endometritis, respectively. Data represent three independent experiments and are presented as the mean ± S.E.M (error bars). Two tailed, student *t*-test, *P < 0.05; **P < 0.01.

Based on a previous study showing increased miR-223 in bovine subclinical endometritis (16), we evaluated its expression level in dairy cows with endometritis. Uteri from Holstein cows obtained from a local slaughterhouse, healthy (*n* = 5) or inflammatory uteri (*n* = 5, with visible lesions), were collected and then subjected to a histological assessment (H&E staining). The results showed that compared with healthy uteri, inflammatory uteri displayed severe inflammation, manifesting as inflammatory cell infiltration, endometrium/glandular epithelial cell detachment, necrosis, myometrium fibrosis, and uterine gland secretions increase (Figure 1A). Some molecular signatures have been found to be associated with subclinical or clinical endometritis in cattle, such as TNF-α and IL-6 (22); our results revealed increased levels of immunostaining for TNF-α in inflammatory uteri (Figure 1B). To further confirm our results, qPCR was used to detect the expression levels of the proinflammatory cytokines IL-1β, TNF-α and IL-6 and showed that these proinflammatory cytokines were significantly upregulated in inflamed uteri (Figure 1C). Next, the miR-223 level was determined by qPCR, and the results showed that there was a higher level of miR-223 in endometritis biospecimens than in those from healthy cows (Figure 1D). These results confirmed that upregulated miR-223 was associated with endometritis in dairy cows.

### Elevated expression of miR-223 following LPS stimulation

**Figure 2:**
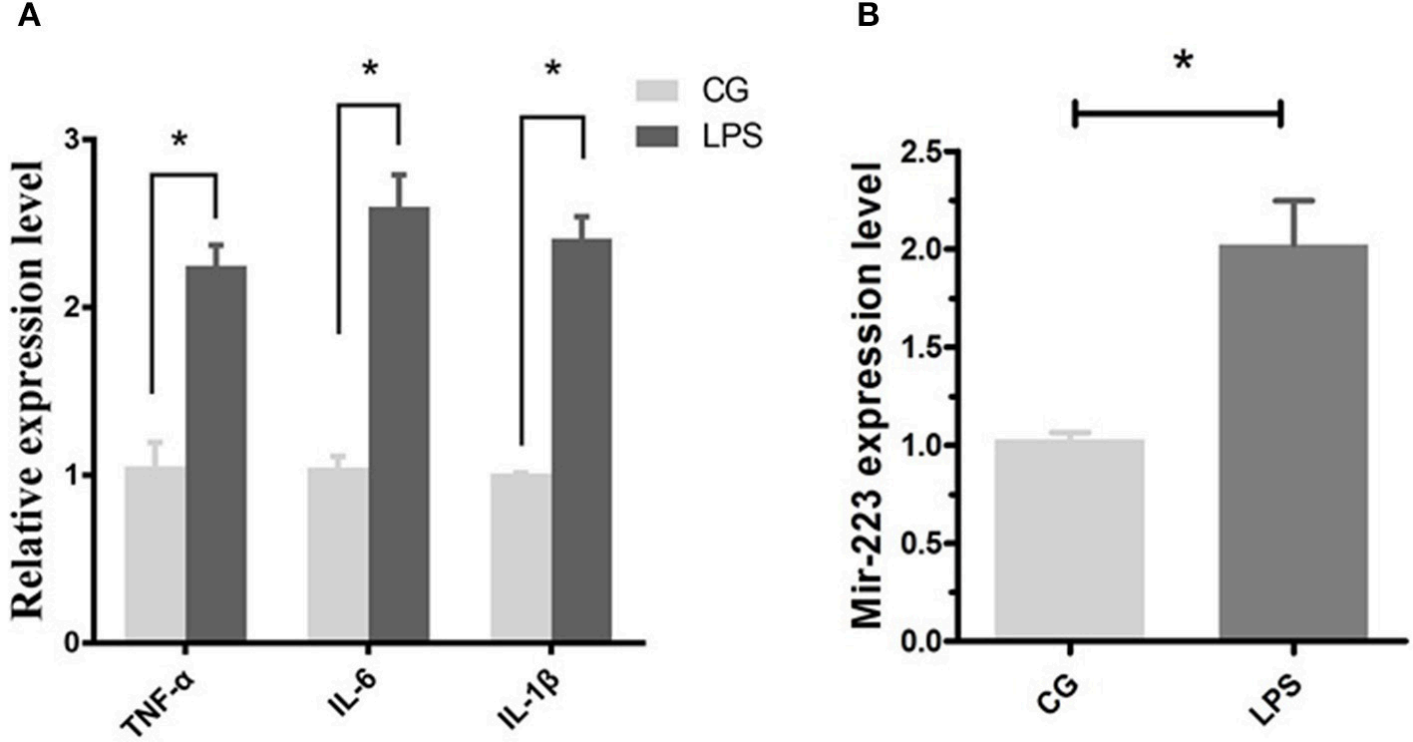
LPS induces miR-223 expression in BEND cells. BEND cells were induced by LPS (0.5 μg/mL) for 2 h, proinflammatory cytokines IL-1β, TNF-α, and IL-6 **(A)** and miR-223 **(B)** were detected by qPCR. CG stands for the control group, LPS stands for the LPS treated group. Data represent three independent experiments and are presented as the mean ± S.E.M (error bars). Two tailed, student *t*-test,*P < 0.05; **P < 0.01.

Bovine endometrial epithelial (BEND) cells were induced by LPS (0.5 μg/mL) for 2 h, and the proinflammatory cytokines IL-1β, TNF-α and IL-6 were significantly upregulated (Figure 2A), accompanied by an increased expression level of miR-223 (Figure 2B). These results indicated that increased miR-223 was induced by LPS in BEND cells.

### NF-κB is required for the induction of miR-223 upon LPS stimulation

**Figure 3:**
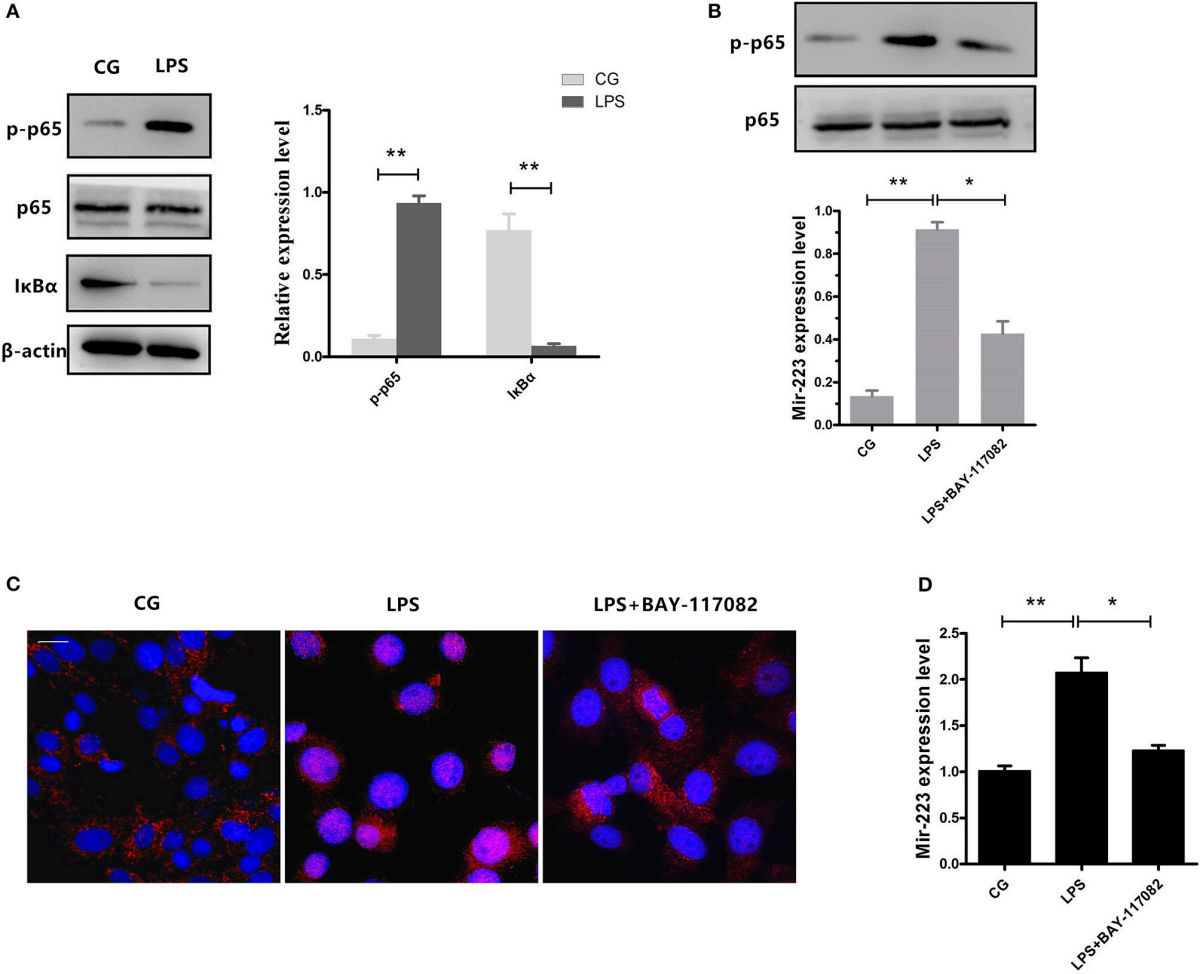
Inhibition of NF-κB activation reduces miR-223 expression. **(A)** BEND cells were induced by LPS (0.5 μg/mL) for 2 h. Western blots were performed to detect the NF-κB p65 and IκBα protein levels. BEND cells were pre-treated with BAY-117082 (20 μM) for 1 h and then exposed to LPS (0.5 μg/ml) for 2 h (LPS+ BAY-117082). **(B)** The NF-κB p65 phosphorylation level was detected by Western blotting; translocation of the p65 subunit from the cytoplasm into the nucleus was evaluated by immunofluorescence **(C)**. Blue spots represent cell nuclei, and red spots represent p-p65 staining; scale bar: 20 μm **(D)**. MiR-223 expression was measured by qPCR. CG stands for the control group, LPS stands for the LPS-treated group. Data represent three independent experiments and are presented as the mean ± S.E.M (error bars). Two tailed, student *t*-test, *P < 0.05; **P < 0.01.

NF-κB is a key regulator in inflammatory processes, and IκBα acts as an inhibitor of NF-κB. Our results showed that phosphorylation of p65 significantly increased, accompanied by a decrease in IκBα expression induced by LPS (Figure 3A). Previous studies have shown that NF-κB can bind to the miR-223 promoter region to promote its transcription (23). To detect whether the increase of miR-223 was caused by the activation of NF-κB, we blocked NF-κB using a specific NF-κB inhibitor. Briefly, cells were pre-treated with BAY-117082 (20 μM) for 1 h and then exposed to LPS (0.5 μg/ml) for 2 h. Inhibition of the translocation of NF-κB from the cytosol to the nucleus was observed, which was also confirmed by Western blotting, showing decreased phosphorylation levels of NF-κB p65 (Figures 3B,C), accompanied by a decrease in miR-223 expression (Figure 3D). These results indicated that LPS-induced miR-223 expression depends on the activation of NF-κB.

### MiR-223 impairs the inflammatory response by attenuating IL-1β expression

**Figure 4:**
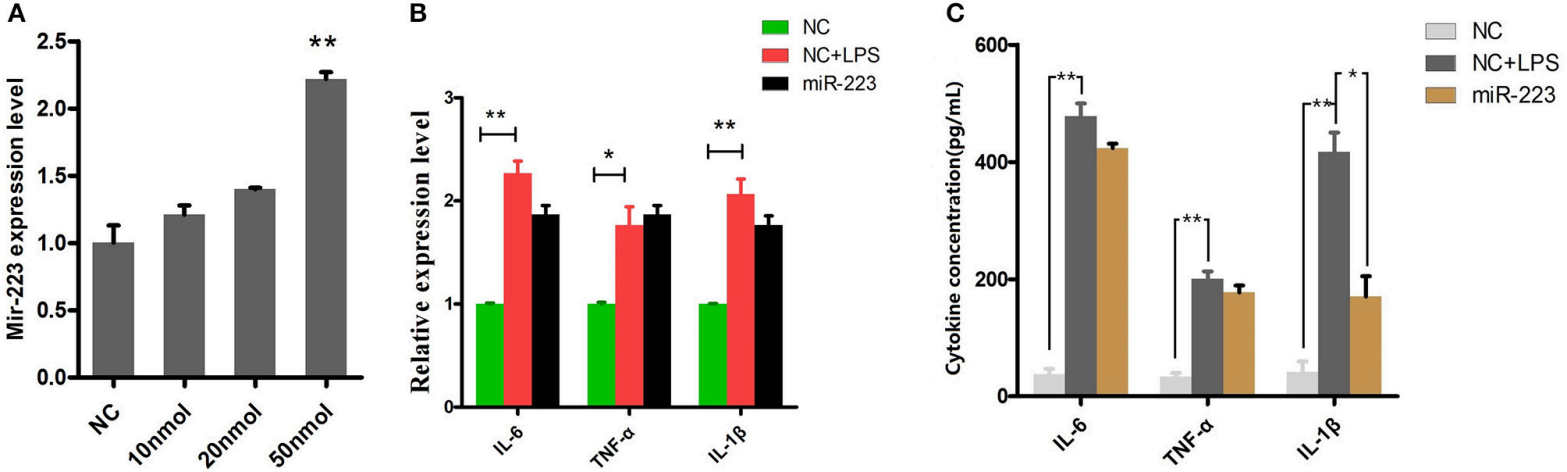
Overexpressing miR-223 reduces LPS-induced IL-1β secretion. BEND cells were transfected with miR-223 mimics (agomiR-223) at different concentrations (0, 10, 20, 50 nmol) for 24 h, and qPCR was used to detect miR-223 expression. **(A)** BEND cells were transfected with miR-223 mimics, followed by 2 h of exposure to LPS (0.5 μg/ml); the proinflammatory cytokines IL-1β, TNF-α, and IL-6 were detected by qPCR **(B)** and ELISA **(C)**. NC stands for cell transfected with the corresponding control oligonucleotide. Data represent three independent experiments and are presented as the mean ± S.E.M (error bars). Two tailed, student *t*-test, *P < 0.05; **P < 0.01.

BEND cells were transfected with miR-223 mimics (agomiR-223) for 24 h (Figure 4A), followed by 2 h of exposure to LPS (0.5 μg/ml), and the proinflammatory cytokines IL-1β, TNF-α and IL-6 were detected by qPCR and ELISA. The results showed that only IL-1β release was significantly inhibited among these LPS-induced proinflammatory cytokines (Figure 4C). However, miR-223 mimics did not affect the mRNA expression of IL-1β, TNF-α and IL-6 (Figure 4B), which indicated that mir-223 selectively inhibited the expression of pro-inflammatory cytokines.

### MiR-223 inhibits IL-1β release by targeting NLRP3

**Figure 5:**
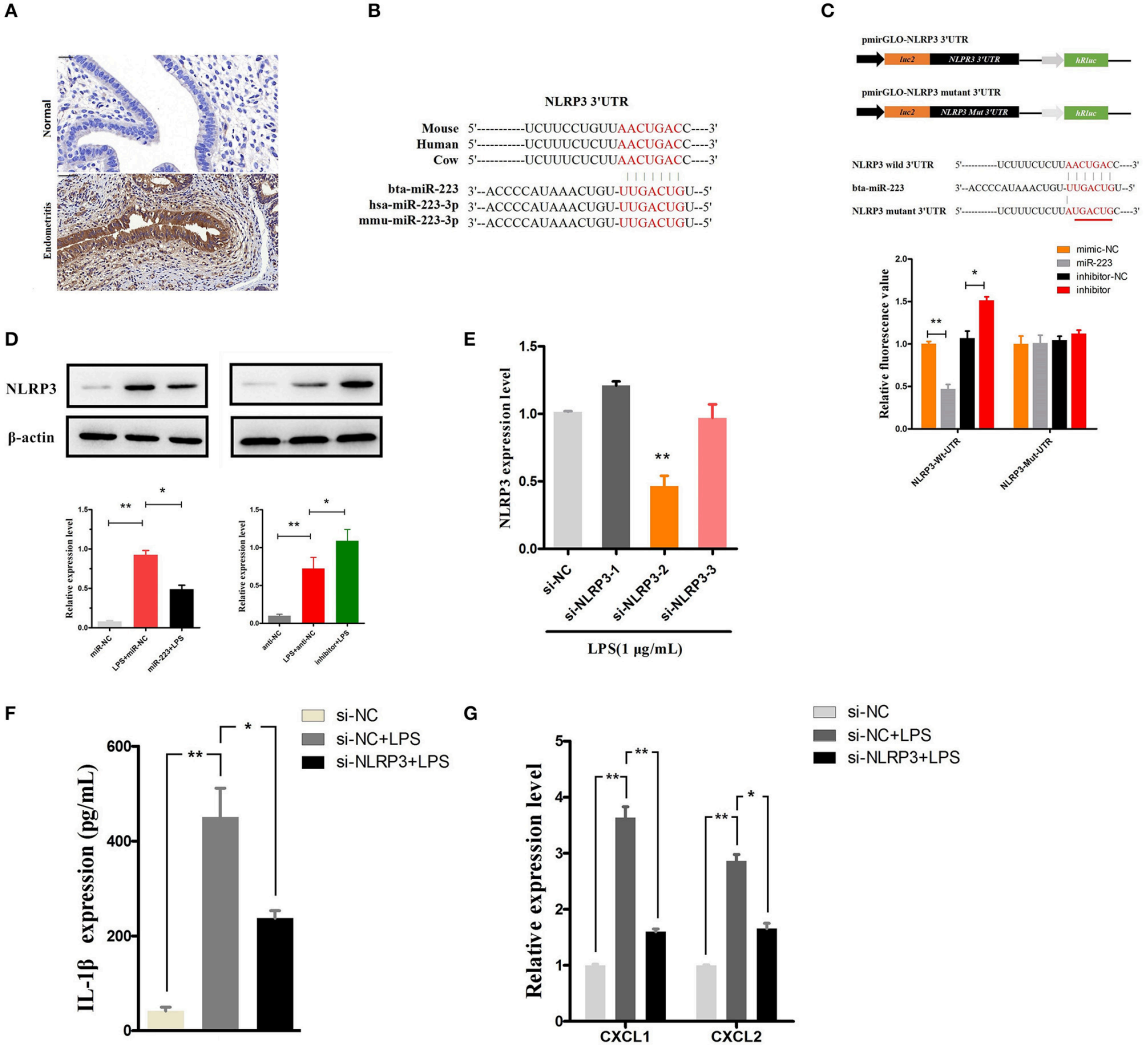
MiR-223 inhibits IL-1β release by targeting NLRP3. **(A)** Immunohistochemical staining of NLRP3, scale bar, 50 μm. **(B)** Conservation of the miR-223 target sequence in NLRP3 among different species (Upper), and conservation of the sequence of miR-223 among different species (Lower). **(C)** Schematic diagram showing dual-luciferase reporter constructs harboring the 3′UTR of NLRP3 with the putative miR-223 binding site. The lower shows the alignment of miR-223 and its target site in the 3′UTR of NLRP3. Six mutated nucleotides of the target site are underlined. 293T cells were cotransfected with agomiR-223, antagomiR-223, or the corresponding control oligonucleotide (mimic-NC, inhibitor-NC) together with a wild-type or mutated NLRP3 3'UTR dual-luciferase reporter plasmid; luciferase activity was normalized by the ratio of firefly and Renilla luciferase signals. **(D)** BEND cells were transfected with agomiR-223, antagomiR-223, or the corresponding control oligonucleotide (miR-NC, anti-NC), and then, the NLRP3 protein levels were determined after 24 h by Western blotting. BEND cells transfected with NLRP3 siRNA or the corresponding control oligonucleotide (si-NC) for 24 h and induced by LPS (1 μg/mL) for 2 h. **(E)** The NLRP3 level was determined by qPCR. **(F)** The concentration of IL-1β in BEND cell supernatant was detected by ELISA. **(G)** The mRNA of chemokines CXCL1, CXCL2 was determined by qPCR. Data represent three independent experiments and are presented as the mean ± S.E.M (error bars). Two tailed, student *t*-test, *P < 0.05; **P < 0.01.

NLRP3, a potential target of miR-223, acts as a key inflammation mediator in many inflammatory diseases by mediating the maturation and secretion of IL-1β (24, 25). Our results also showed an increased level of NLRP3 during endometritis in dairy cows (Figure 5A). The binding sites of bta-miR-223 conserved across species are shown in Figure 5B, and dual luciferase activity assays showed that miR-223 mimic transfection significantly decreased the luciferase activity of the NLRP3 3′UTR group but was relatively enhanced by the inhibition of miR-223 (Figure 5C). The inhibitory effects of miR-223 on luciferase activity were clearly lost when binding sites were absent (Figure 5C). The NLRP3 protein was also assessed in BEND cells transfected with miR-223 mimics (agomiR-223) or inhibitors (antagomir-223). NLRP3 protein expression was significantly suppressed by overexpression of miR-223, whereas inhibition of miR-223 increased NLRP3 expression (Figure 5D). These results suggest that miR-223 negatively regulates the expression of NLRP3 in BEND cells by targeting NLRP3. To confirm the NLRP3-inflammasome mediated release of IL-1β, blockade of the NLRP3 inflammasome (Figure 5E) using a specific siRNA (si-NLRP3) showed a decreased release of IL-1β and the mRNA of the IL-1β-inducible chemokines CXCL1 and CXCL2 (26) (Figures 5F,G). However, blockade of the NLRP3 inflammasome did not affect the mRNA expression of IL-1β, TNF-α, and IL-6 (Supplementary Figure 1). Altogether, these results suggest that miR-223 impairs the inflammatory response by inhibiting the NLRP3 inflammasome and its mediated IL-1β secretion.

### Inhibition of miR-223 exacerbates LPS-induced endometritis

**Figure 6:**
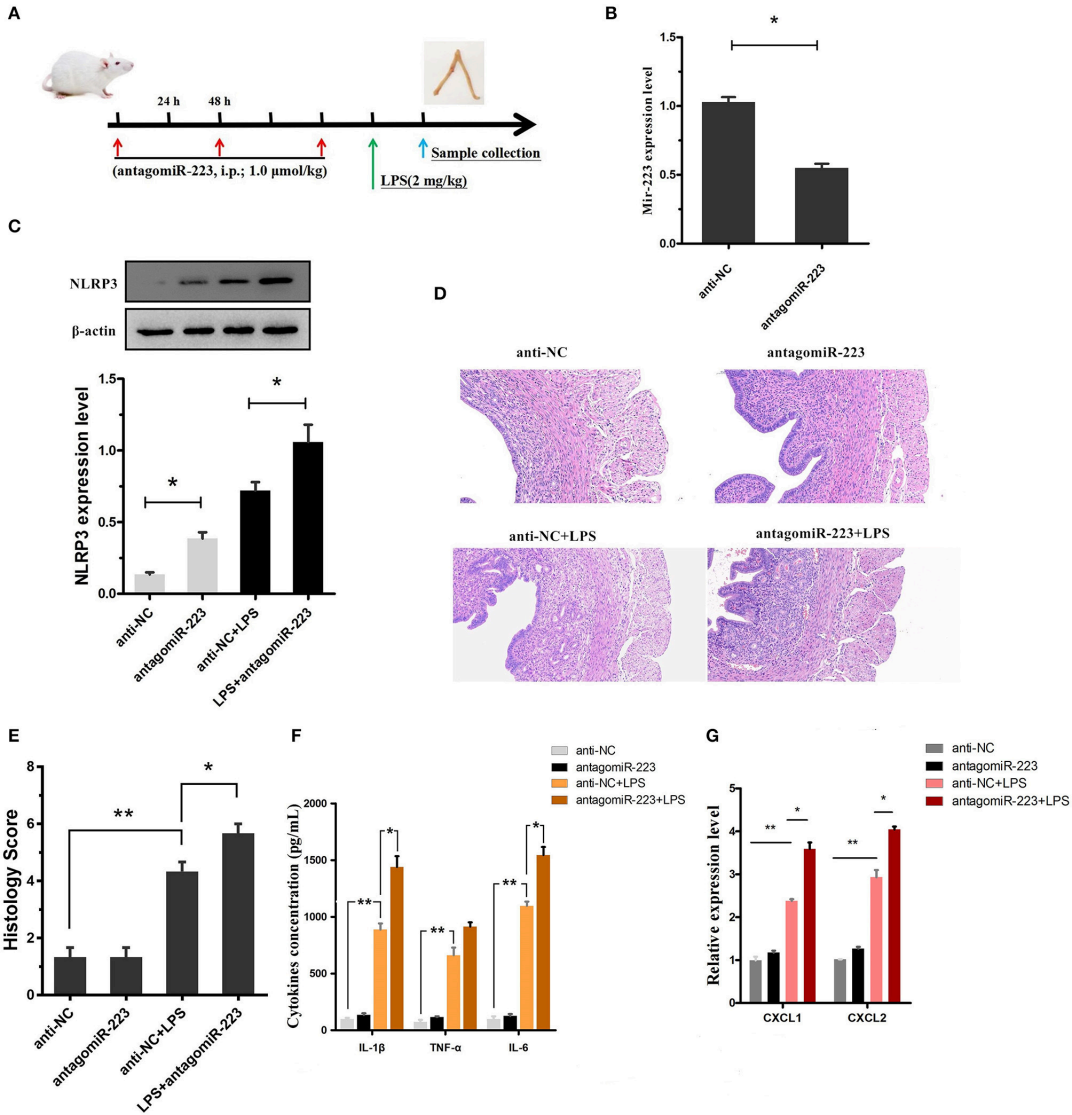
Inhibition of miR-223 enhances susceptibility to LPS-induced endometritis. **(A)** Time axis of experimental animal treatment. **(B)** MiR-223 expression was measured in uterine tissues by qPCR. **(C)** The NLRP3 protein level was assessed by Western blotting. **(D)** H&E staining of uterine tissue, scale bar, 50 μm. **(E)** Histopathology scores. **(F)** ELISA measurement of IL-1β, TNF-α, and IL-6 in whole uterine biopsies. **(G)** Relative mRNA of CXCL1 and CXCL2 was assessed by qPCR in whole uterine biopsies; *n* = 3–5 mice/group. Data represent three independent experiments and are presented as the mean ± S.E.M (error bars). Statistical significance determined by unpaired Student's *t*-test. *P < 0.05; **P < 0.01.

Our *in vitro* experiments implicated miR-223 as a critical negative regulator of NLRP3, leading to the alleviation of LPS-induced inflammation. Next, to confirm the effect of miR-223 deficiency on LPS-induced endometritis, miR-223 inhibitor (antagomiR-223) was used to transiently attenuate the miR-223 level *in vivo* (i.p.; 1.0 μmol/kg; once every 48 h, a total of 3 times), followed by administration of LPS (2 mg/kg, 24 h after the last injection of miR-223 inhibitor) into the uterine cavity to induce acute uterine injury or endometritis in mice (Figure 6A). Our data revealed that the miR-223 level was increased by LPS treatment and decreased by pre-treated miR-223 inhibitors (Figure 6B). Moreover, transient inhibition or reduction of miR-223 expression exacerbated endometriosis, as indicated by the histological indices, increased NLRP3, IL-1β, IL-6, and TNF-α secretion, as well as the IL-1β-inducible chemokines CXCL1 and CXCL2 mRNA transcripts (Figures 6C–G). These results suggested that miR-223 acted as an essential anti-inflammatory factor in inflammation disorders and protected the uterus against inflammatory damage.

### Delivery of miR-223 mimics constrains experimental LPS-induced endometritis

**Figure 7:**
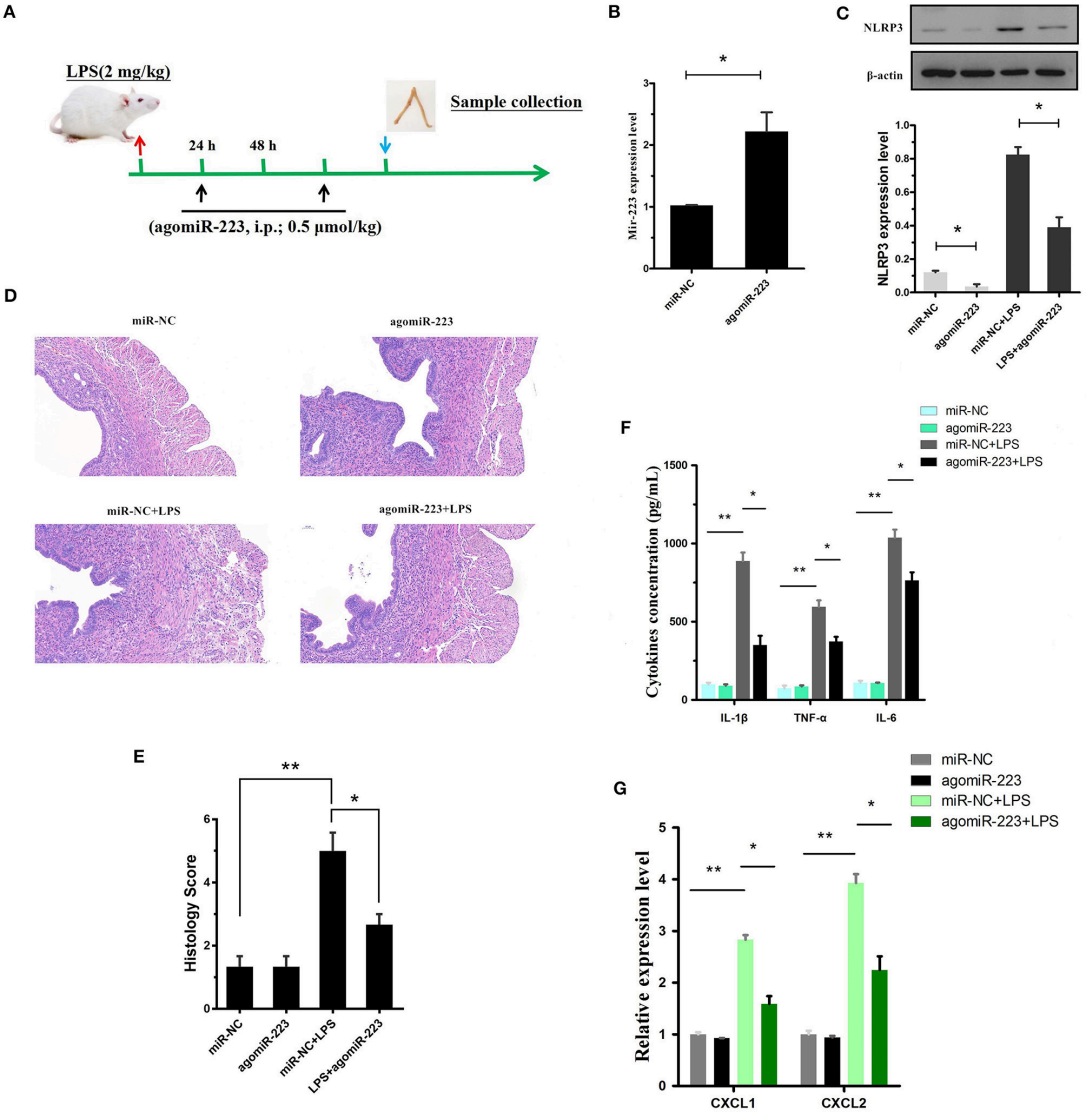
Delivery of the mir-223 mimics attenuates experimental LPS-induced endometritis. **(A)** Time axis of experimental animal treatment. **(B)** MiR-223 expression was measured in uterine tissue by qPCR. **(C)** The NLRP3 protein level was assessed by Western blotting. **(D)** H&E staining of uterine tissue, scale bar, 50 μm. **(E)** Histopathology scores. **(F)** ELISA measurement of IL-1β, TNF-α, and IL-6 in whole uterine biopsies. **(G)** Relative mRNA of CXCL1 and CXCL2 was assessed by qPCR in whole uterine biopsies; *n* = 3–5 mice/group. Data represent three independent experiments and are presented as the mean ± S.E.M (error bars). Statistical significance determined by unpaired Student's *t*-test. *P < 0.05; **P < 0.01.

To verify the therapeutic potential of miR-223 *in vivo*, mouse endometritis was induced by LPS and then treated with miR-223 mimics (i.p., 0.5 μmol/kg, on day 1 and 3 after LPS administration, Figure 7A). As shown in Figure 7B, therapeutic intervention with synthetic miR-223 mimics produced a significant increase in uterine miR-223 expression, accompanied by a marked protection from LPS-induced endometritis, as indicated by the attenuated pathology conditions (Figures 7D,E). Furthermore, mmu-miR-223 mimic treatment repressed the expression of NLRP3 (Figure 7C) and increased the repression of IL-1β, IL-6, TNF-α, and CXCL1, CXCL2 mRNA transcripts (Figures 7F,G). Collectively, these data confirmed the pivotal role of miR-223 during endometritis and represented a principled evidence-based treatment study of the use of miRNA mimics for the treatment of experimental endometritis.

## Discussion

Endometritis caused by various aerobic, anaerobic, Gram-positive and Gram-negative bacteria, including subclinical and clinical endometritis, is known to affect the milk production and reproductive performance of dairy cows by inducing uterine inflammation, leading to a serious economic burden. During bacterial infections, the endometrium responds to foreign invaders by activating or suppressing certain biochemical or molecular signals. Therefore, better understanding these molecular events may lead to important molecular diagnostic markers for the development of subclinical and/or clinical endometritis and provide potential therapeutic targets. LPS is the main component of Gram-negative bacterial cell walls, and LPS-induced endometritis or uterine injury exhibits severe endometrial damage and is thus used to mimic these uterine disorders to evaluate the potential protection of a drug *in vivo* (6). miR-223 is known to be a myeloid-enriched anti-inflammatory microRNA that migrates to sites of inflammation through myeloid cells, such as neutrophils and monocytes, causing local miR-223 levels to be significantly upregulated to exert anti-inflammatory effects. In fact, in addition to myeloid cells, resident cells, such as various types of epithelial cells, also play important roles in the inflammatory process. Zhou et al. showed that miR-223 in both phagocytes and epithelial cells cooperates to restrict the magnitude of inflammation (27). Our results revealed that activation of NF-κB in LPS-induced BEND cells *in vitro* and endometritis *in vivo* promoted the transcription of miR-223 and thus inhibited the activation of inflammatory mediators of NLRP3-mediated IL-1β production to protect the uterus from inflammatory damage. Our study reconfirms that miR-223 from indigenous cells is involved in the process of inflammation protection.

miRNAs are implicated in various biological processes and ae believed to be useful as diagnostic markers of multiple forms of inflammatory diseases and cancer (28, 29). Hailemariam and Salilew-Wondim et al. revealed a large number of miRNA profiles associated with clinical and/or subclinical endometritis of dairy cows by RNA-seq technology (17, 30), suggesting that miRNAs also play an important role in endometritis in cattle. However, their underlying molecular mechanisms or signaling pathways remain unclear. Previous studies revealed that miR-223 mutant mice spontaneously develop inflammatory lung pathology and exhibit exaggerated tissue destruction (31) and that miR-223 regulated macrophage polarization and protected mice from adipose tissue inflammation (32); in addition, altered expression of miR-223 has been linked to several immune disorders, including rheumatoid arthritis (33) and type 2 diabetes mellitus (34). Our study showed a similar result: increased miR-223 induced by LPS stimulation both *in vitro* and *in vivo*, which acted to fine-tune endometritis.

The NLRP3 inflammasome, which is composed of NLRP3, ASC, and caspase-1, has been implicated in the pathogenesis of a variety of inflammatory diseases (35). The NLRP3 inflammasome can sense many different factors, such as LPS, and upon activation, the assembly of the inflammasome results in the cleavage and activation of caspase-1, with constitutive IL-1β production resulting in serious inflammatory disorders (36), including type 2 diabetes (T2D), gout, atherosclerosis, and neurodegenerative diseases (37–39).Therefore, activation of the inflammasome needs to be strictly controlled to prevent excessive inflammation. However, the expression level of NLRP3 has been shown to be a limiting step for inflammasome activation (40). Thus, discovering a novel molecule inhibitor that targets the inflammasome sensor NLRP3 offers a new path for the development of selective inflammasome blockers with potential therapeutic benefits in a wide range of human diseases (41). Our *in vitro* experiments showed that miR-223 negatively regulated NLRP3 expression through a conserved binding site within the 3′ untranslated region of NLRP3 and therefore functioned as an important rheostat that controls NLRP3 inflammasome activity, which was consistent with a previous study (42). Meanwhile, short-term reduction of miR-223 expression with miR-223 inhibitors enhanced susceptibility to LPS-induced endometritis, and intervention with miR-223 mimics led to a reduction in NLRP3 production that translated into a decreased capacity to generate biologically active IL-1β, thus increasing the susceptibility to those genital tract microorganisms, which are especially susceptible to destruction following the induction of these pro-inflammatory cytokines/chemokines. Moreover, use of NLRP3-specific interfering RNA (si-NLRP3) also attenuated LPS-induced inflammatory responses *in vitro*. These results suggested that NLRP3 can be used as a potential target for the treatment of endometritis and that miR-223 may be able to be used as a small molecule inhibitor in the clinical treatment of NLRP3-related diseases. Interestingly, the utility of treatment with mir-223 mimics has been demonstrated in experimental colitis (9).

**Figure 8:**
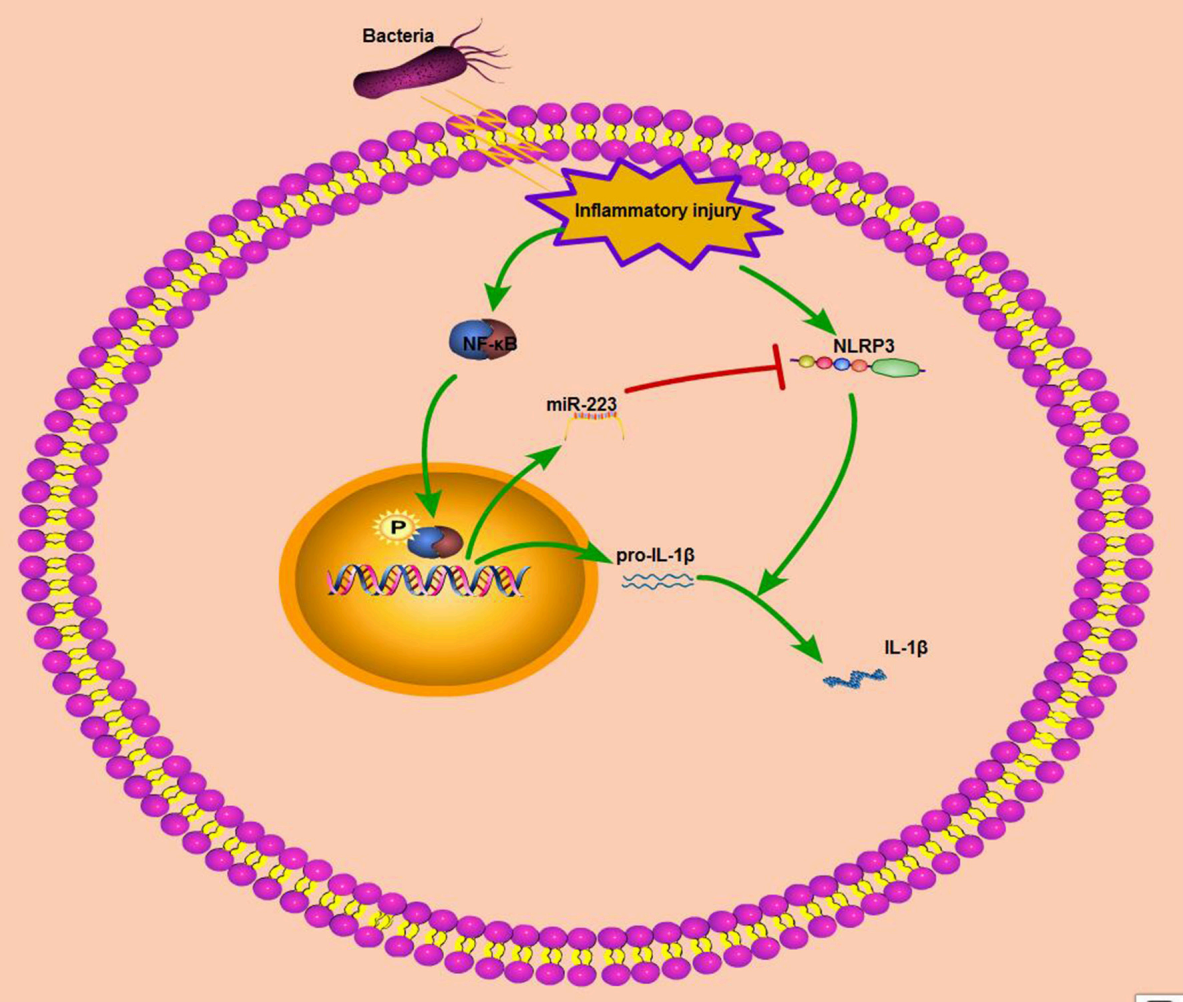
Schematic diagram of mir-223 regulates endometritis by inhibiting NLRP3 activation. During endometritis, bacterial infection activates NF-κB to promote the transcription of pro-IL-1β and miR-223. In addition, NLRP3 inflammasome activation also occurs during endometritis; the activation of NLRP3 promotes the maturation of IL-1β and exacerbates the inflammatory response. MiR-223, by inhibiting the expression of NLRP3, attenuates the secretion of proinflammatory cytokine IL-1β and improves endometritis.

Nuclear factor-κB (NF-κB), is another archetypal molecular driver of the inflammatory response (43). LPS belongs to a prototypical class of pathogen-associated molecular patterns (PAMPs), and following the recognition of PAMPs, LPS induces a downstream signaling cascade and activates NF-κB signaling (44). IκBα is a known inhibitor of NF-κB, and once activated, IκBα is degraded and the NF-κB subunit p65 translocates from the cytoplasm to the nucleus, which induces the transcription of downstream inflammatory genes, such as IL-1β, TNF-α, and IL-6; enhanced proinflammatory cytokines drive robust inflammatory responses, and these cytokines are tightly regulated at multiple levels to avoid excessive tissue damage (45). Thus, inhibition of the release of inflammatory cytokines may be a target for anti-inflammatory drug therapies. Our *in vivo* and *in vitro* results showed that miR-223 decreased the production of these inflammatory cytokines, although the *in vitro* inhibitory effect was not very significant possibly because of the ability of miR-223 to regulate macrophage polarization *in vivo* (32). In addition, miR-223 has been identified as the NF-κB-sensitive miRNA that is regulated by NF-κB and targets signaling proteins of innate immune responses (20). Once NF-κB is activated, miR-223 is also upregulated due to NF-κB binding to the miR-223 promoter region (23). Our study showed that increased phosphorylation levels of NF-κB p65 were accompanied by elevated miR-223 in both endometritis and LPS-induced BEND cells; however, blockade of NF-κB downregulated the miR-223 expression significantly. Interestingly, Zhou et al. recently demonstrated that overexpression of miR-223 suppressed the canonical NF-κB pathway in epithelial cells (27). Our results showed that miR-223 attenuated the production of pro-inflammatory cytokines, which was induced by the activation of the canonical NF-κB pathway. That is, activation of NF-κB promoted intracellular mir-223 levels, which, when they reached a certain level, inhibited NF-κB activity and impaired inflammatory processes. Although there was no direct evidence in this study that mir-223 inhibited the activation of NF-κB (data not shown), mir-223 expression may be spatiotemporally dependent on NF-κB activity. Altogether, these results suggest that during endometritis, the body will actively release resistance signals, such as activation of NF-κB, on the one hand to promote the release of pro-inflammatory cytokines or chemokines, which recruit immune cells to the injured site to help tissue repair; on the other hand, activation of NF-κB can promote the transcription of some anti-inflammatory molecules, including miR-223, that target NLRP3 and inhibit the activation of the NLRP3 inflammasome and its induced release of pro-inflammatory cytokines or chemokines, resulting in an improved inflammatory condition (Figure 8). However, due to experimental limitations, it was not possible to verify the role of NF-κB in cattle in this study. Although both miR-223 and NLRP3 are highly conserved across species, more research is needed to verify the clinical therapeutic capabilities of miR-223.

Collectively, our study highlights that miR-223 serves to constrain the level of NLRP3 activation and serves as a protective factor in the inflammatory response. Thus, pharmacologic stabilization of miR-223 may hold promise as a future novel therapeutic modality for active flares of endometritis and other inflammatory diseases in cows.

## Author contributions

GZ and GD conceived of and designed the experiments. GZ, TZ, HW, KJ, and YY performed the experiments. GZ, TZ, CQ, and GD analyzed the data. GZ, GD, TZ, YY, and AS wrote the paper. All of the authors read and approved the final manuscript.

### Conflict of interest statement

The authors declare that the research was conducted in the absence of any commercial or financial relationships that could be construed as a potential conflict of interest.
