# Supplementary material for: The Potential Therapeutic Role of miR-223 in Bovine Endometritis by Targeting the NLRP3 Inflammasome
Source: Front Immunol. 2018 Aug 22;9:1916. doi: 10.3389/fimmu.2018.01916 (PMC6113393; doi:10.3389/fimmu.2018.01916)
Supplement: Supplementary file 1 [file Data_Sheet_1.DOC]

| miRNA ID | Accession Number | Primer sequence (5'-3') |
| --- | --- | --- |
| bta-miR-223 | MIMAT0009270 | UGUCAGUUUGUCAAAUACCCCA |
| mmu-miR-223-5p | MIMAT0000665 | UGUCAGUUUGUCAAAUACCCCA |
|  | RT-primer | CTCAACTGGTGTCGTGGAGTCGGCAATTCAGTTGAGTGGGGTAT |
|  | F： | TCGGCAGGTGTCAGTTTGTCAA |
|  | R： | CTCAACTGGTGTCGTGGAGT |
| U6 | F： | CTCGCTTCGGCAGCACATATACT |
|  | R： | ACGCTTCACGAATTTGCGTGTC |

**Supplementary table 1 miRNA primers design**

**Supplementary table 2oligonucleotide primers used for qPCR**.

| Species | Name | Accession Number | Primer sequence (5'-3') | Product size |
| --- | --- | --- | --- | --- |
|  | CXCL-1 | NM_008176.3 | F:CATGGCTGGGATTCACCTCA | 128 bp |
|  |  |  | R:GAGCTTCAGGGTCAAGGCAA |  |
|  | CXCL-2 | NM_009140.2 | F:GCGGTCAAAAAGTTTGCCTTG | 80 bp |
|  |  |  | R:AGCCTTGCCTTTGTTCAGTATC |  |
|  | IL-1β | NM_008361.4 | F:CCTGGGCTGTCCTGATGAGAG | 131 bp |
|  |  |  | R:TCCACGGGAAAGACACAGGTA |  |
|  | TNF-α | NM_013693.3 | F:CTTCTCATTCCTGCTTGTG | 198 bp |
|  |  |  | R:ACTTGGTGGTTTGCTACG |  |
|  | IL-6 | NM_031168.1 | F:GGCGGATCGGATGTTGTGAT | 199 bp |
|  |  |  | R:GGACCCCAGACAATCGGTTG |  |
|  | GAPDH | NM_001289726.1 | F:TGTTTCCTCGTCCCGTAG | 108 bp |
|  |  |  | R:CAATCTCCACTTTGCCACT |  |
| Bovine | NLRP3 | NM_001102219.1 | F:TGGCTGTAACATTCGTAGA | 174 bp |
|  |  |  | R:AATAGATGCCTCAGTCCC |  |
|  | CXCL-1 | NM_175700.2 | F:GGAAGTGTGTCTCAACCCCA | 79 bp |
|  |  |  | R:TCAGTTGGCACTAGCCTTGTT |  |
|  | CXCL-2 | NM_174299.3 | F:AATGCTGCTCCTGCTCCTG | 173 bp |
|  |  |  | R:TGGCTATGACTTCGGTTTGG |  |
|  | IL-1β | NM_174093.1 | F:AAAAATCCCTGGTGCTGGCT | 195 bp |
|  |  |  | R:GGGTGGGCGTATCACCTTTT |  |
|  | TNF-α | NM_173966.3 | F:CTCCTTCCTCCTGGTTGCAG | 92 bp |
|  |  |  | R:CACCTGGGGACTGCTCTTC |  |
|  | IL-6 | NM_173923.2 | F:CTACCTCCAGAACGAGTATG | 136 bp |
|  |  |  | R:CAGCAGGTCAGTGTTTGTGG |  |
|  | GAPDH | NM_001034034.2 | F:GGTCACCAGGGCTGCTTT | 128 bp |
|  |  |  | R:CTGTGCCGTTGAACTTGC |  |

**Supplementary table 3siRNA sequence design**

| SiRNA | Sequence (5'-3') |
| --- | --- |
| si-NLRP3-1 | CCUGGAAGACAUAGACUUUTT |
|  | AAAGUCUAUGUCUUCCAGGTT |
| si-NLRP3-2 | GCGAGAAAUUCUACAGCUUTT |
|  | AAGCUGUAGAAUUUCUCGCTT |
| si-NLRP3-3 | CCACACUUCUAACUUCUAATT |
|  | UUAGAAGUUAGAAGUGUGGTT |
| si-NC | UUCUCCGAACGUGUCACGUTT |
|  | ACGUGACACGUUCGGAGAATT |

**Supplementary figure 1**


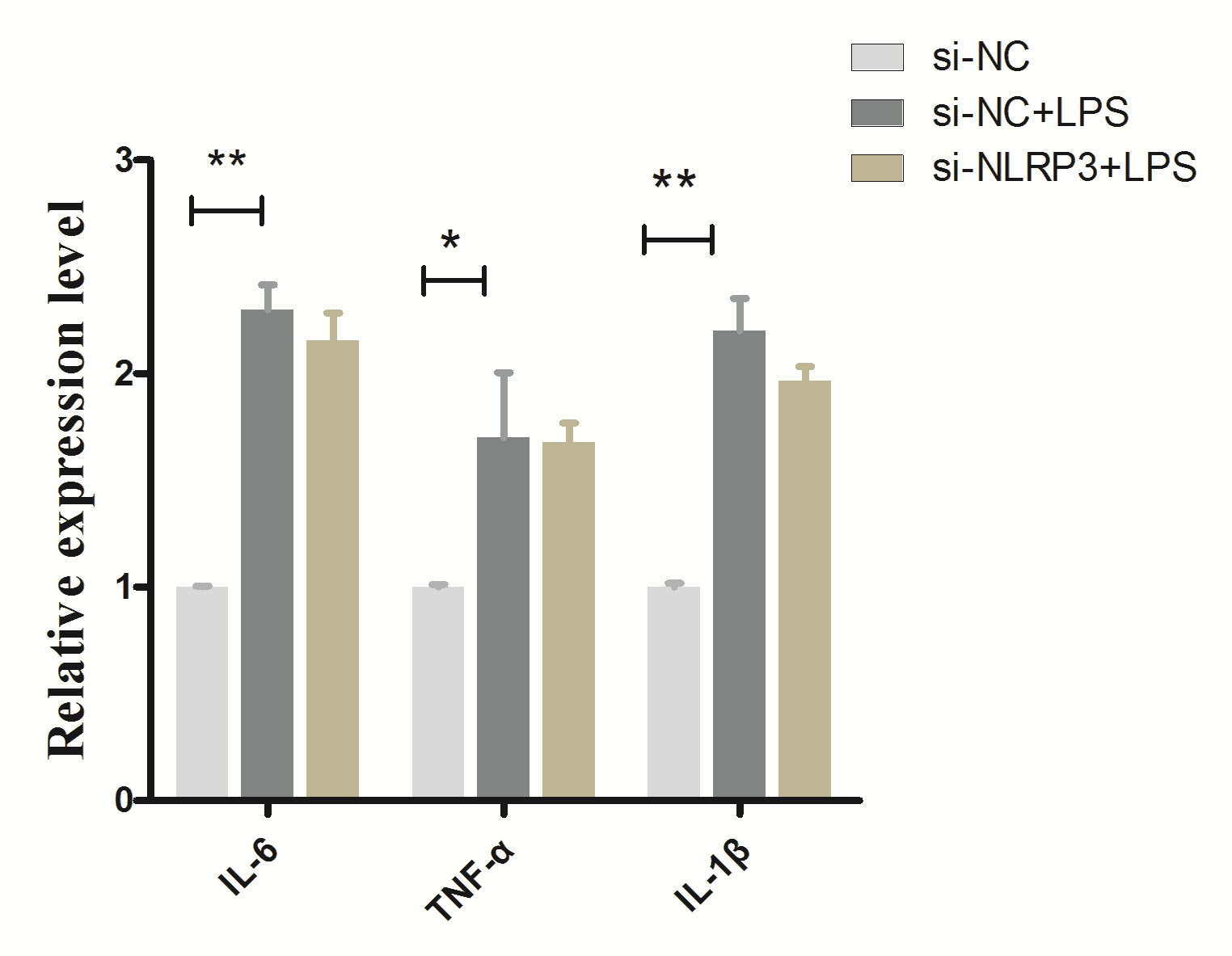


**Figure S1** The effect of blocking NLRP3 on the mRNA level of TNF-α, IL-1β, and IL-6 in LPS-inducedBEND cells. Blocked NLRP3 inflammasomeusing specific siRNA (si-NLRP3)and then stimulated with LPS (0.5 μg/mL) for 2 h. Si-NC(siRNA negative control). Data representsthree independent experiments and are presented as the mean±S.E.M (error bars). Two tailed, student t-test,*P<0.05; **P<0.01.
